# Exploring *Ochradenus baccatus*: A Novel Source of Bioactive Compounds and Phytochemical Insights for Uncharted Therapeutic Applications

**DOI:** 10.3390/life15091448

**Published:** 2025-09-16

**Authors:** Salma Saddeek

**Affiliations:** Department of Chemistry, Faculty of Sciences, University of Hafr Al Batin, Hafr Al Batin 39524, Saudi Arabia; salmayms@uhb.edu.sa

**Keywords:** *Ochradenus baccatus*, Alzheimer’s disease, phytochemicals, phenolics, flavonoids, antioxidant activity, AChE inhibition, amyloid-beta, neuroprotection, HPLC, GC-MS

## Abstract

*Ochradenus baccatus* (*O. baccatus*), a resilient medicinal plant native to arid regions, was systematically investigated for its neuroprotective potential against Alzheimer’s disease. Comprehensive phytochemical profiling of different plant parts revealed that the leaves possessed the highest levels of total phenolics (67.8 mg GAE/g) and flavonoids (49.2 mg QE/g), correlating with strong antioxidant activity (DPPH IC_50_ = 19.8 µg/mL, FRAP = 832 µmol Fe^2+^/g). HPLC and GC-MS analyses identified multiple bioactive flavonoids and fatty acids. The leaf extract demonstrated potent in vitro AChE inhibition (IC_50_ = 32.5 µg/mL) and significantly reduced amyloid-β aggregation (by 50%). In vivo, it ameliorated cognitive deficits in scopolamine-induced mice, as evidenced by improved performance in Morris Water Maze and Y-maze tests, and restored hippocampal neuronal density (CA3: +29.7%, DG: +30%). These findings highlight the therapeutic promise of *O. baccatus* leaves as a rich source of multifunctional anti-Alzheimer’s phytochemicals.

## 1. Introduction

*Ochradenus baccatus* Delile (family Resedaceae) is a perennial xerophytic shrub native to the arid and semi-arid regions of the Middle East, North Africa, and the Arabian Peninsula. It is commonly found in gravelly plains and rocky outcrops, particularly in Saudi Arabia, Jordan, Egypt, and parts of the Levant [1,2,3,4,5,6]. Morphologically, the plant is characterized by slender green stems, linear to lanceolate leaves, and small yellowish flowers, with fleshy red berries that represent the fruiting part. Traditionally, different parts of *O. baccatus*, particularly the leaves and fruits, have been used in folk medicine for the treatment of infections, rheumatism, skin inflammation, and gastrointestinal disturbances [7].

Phytochemical investigations into *O. baccatus* have confirmed the presence of bioactive secondary metabolites, notably phenolics, flavonoids, saponins, and triterpenoids [8,9]. High-performance liquid chromatography (HPLC), gas chromatography–mass spectrometry (GC-MS), and nuclear magnetic resonance (NMR) analyses have identified compounds such as rutin, kaempferol, quercetin, lupeol, β-sitosterol, and various fatty acid derivatives [10,11,12,13]. These constituents are well-documented for their antioxidant, anti-inflammatory, and neuroprotective properties in other botanical systems [7,14,15]. Historically, the therapeutic relevance of *O. baccatus* has been supported by in vitro and in vivo studies highlighting its antibacterial, antidiabetic, cytoprotective, and hepatoprotective effects [16]. More recently, the plant has drawn attention in neuropharmacological research due to the presence of flavonoid-rich fractions that interact with cholinergic, antioxidative, and amyloidogenic pathways—mechanisms critically implicated in Alzheimer’s disease (AD). AD is a progressive neurodegenerative disorder characterized by memory impairment, cognitive decline, oxidative damage, cholinergic dysfunction, and amyloid-β plaque accumulation in the brain [17]. Despite advances in pharmacotherapy, current treatments offer symptomatic relief with limited disease modifying potential and often incur adverse effects. Consequently, there is an increasing emphasis on identifying multi-targeted, naturally derived agents capable of modulating several neurodegenerative pathways concurrently [18,19]. Polyphenol- and flavonoid-rich plant extracts are particularly promising due to their demonstrated antioxidant activity, acetylcholinesterase inhibition, and anti-amyloid properties [20,21].

In this context, *O. baccatus* emerges as a valuable candidate for exploration, given its unique phytochemical composition and traditional medicinal relevance. However, the neuropharmacological properties of this plant, especially its potential role in AD mitigation, remain under-investigated. Prior evidence on the antioxidative and anti-inflammatory roles of its extracts warrants a comprehensive biochemical and pharmacological analysis focused on memory-related endpoints and neuropathological biomarkers.

## 2. Materials and Methods

### 2.1. Plant Material Collection and Authentication

*O. baccatus* was collected from Sinai: Wadi Feiran, mountainous regions of South Sinai (Egypt) during the flowering season (March–April 2025). Botanical identification was confirmed by Department of Botany, Cairo University. A voucher specimen (No. PE-GTRO-142/2025) was deposited at the university herbarium. Plant organs (leaves, roots, fruits, stems) were separated, shade-dried (25 °C), and pulverized (≤1 mm particle size) using an industrial grinder (Retsch SM 200, Haan, Germany).

### 2.2. Extract Preparation

Dried powder (100 g per organ) underwent reflux extraction (3 h, 60 °C) with 80% ethanol (1:10 *w*/*v*). The filtrate was concentrated (Rotary Evaporator Büchi R-300, Postfach, Switzerland) and lyophilized (Free Zone 4.5, Labconco, Fullerton, CA USA) to yield crude extracts. For biological assays, extracts were dissolved in 0.1% DMSO (Sigma-Aldrich, Taufkirchen, Germany) [22].

### 2.3. Phytochemical Analysis

#### 2.3.1. Quantitative Profiling

- Total Phenolic Content (TPC): Folin–Ciocalteu method. Absorbance at 765 nm. Results as mg gallic acid equivalents/g extract (mg GAE/g) [23].
- Total Flavonoid Content (TFC): Aluminum chloride method. Absorbance at 510 nm. Results as mg quercetin equivalents/g extract (mg QE/g) [24].
- HPLC-DAD: Shimadzu LC-20AD; C18 column (4.6 × 250 mm, 5 μm). Gradient: 0.1% formic acid (A) and acetonitrile (B) (0–35 min, 10–95% B). Peaks quantified against standards (rutin, quercetin, kaempferol, lupeol) (Sigma-Aldrich) [25].
- GC-MS: Agilent 7890B/5977A MSD; HP-5MS column (30 m × 0.25 mm). Derivatization: BSTFA + 1% TMCS (70 °C, 1 h). Compounds identified via NIST 2020 library (match factor > 85%) [26].

#### 2.3.2. Antioxidant Assays

- DPPH Radical Scavenging: IC_50_ calculated from dose–response curves [27].
- ABTS^+^ Decolorization: Trolox equivalent antioxidant capacity (TEAC) [28].
- FRAP: Ferric reduction potential [29].

### 2.4. In Vitro Biological Assays

#### 2.4.1. Anti-Inflammatory Activity

- COX-1/COX-2 inhibition assessed via ELISA (Cayman Chemical Kit #701050, Ann Arbor, MI, USA) [30].
- Extract concentration: 100 µg/mL. Selectivity Index = (COX-2 %Inh)/(COX-1 %Inh).

#### 2.4.2. Anti-Alzheimer Activity

- AChE Inhibition: Ellman’s method [31]. IC_50_ determined using donepezil (Sigma D6821, Taufkirchen, Germany) as positive control.
- Amyloid-β Aggregation: Thioflavin T fluorescence assay [32].

### 2.5. In Vivo Studies

#### 2.5.1. Animal Ethics

Male BALB/c mice (25–30 g; n = 10/group) were housed under standard conditions (12 h light/dark, 25 °C). The study followed ethical standards for animal research. Approval for the use of laboratory animals in experimental procedures was granted by the Standing Committee on Research Ethics at Cairo University. The protocol received approval under the number SCREA-CU. 426/2025, with the Review Board’s endorsement dated 16 March 2025.

#### 2.5.2. Alzheimer’s Model Induction and Treatment

- Scopolamine Model: Mice received scopolamine hydrobromide (1 mg/kg/i.p.) daily for 14 days [33].
- Treatment Groups: Group 1: Control (saline), Group 2: Scopolamine (1 mg/kg), Group 3: Scopolamine + *O. baccatus* leaf extract (200 mg/kg/p.o.)

#### 2.5.3. Behavioral Tests

- Morris Water Maze (MWM): Escape latency (acquisition, 5 days) and probe trial (target quadrant time) recorded. Pool diameter: 120 cm; platform: 10 cm [34].
- Y-Maze: Spontaneous alternation (%) and total arm entries [35].

#### 2.5.4. Histopathology

Mice were perfused (4% paraformaldehyde). Brains were sectioned (coronal, 10 μm) and stained:

- Neuronal Density: Cresyl violet (CA3/DG subregions) [36,37].
- Amyloid-β Plaques: Congo red [38].

Images analyzed via Image J 1.53 k (cells/mm^2^).

### 2.6. Statistical Analysis

Data expressed as mean ± SD. One-way ANOVA followed by Tukey’s HSD (SPSS v27.0). Significance: *p* < 0.05. Correlation analyses used Pearson’s coefficient.

## 3. Results

### 3.1. Phytochemical Quantification

Phytochemical quantification such as phenolic and flavonoid content in Table 1 shows that TPC varied significantly across different parts of *O. baccatus*, with the highest concentration observed in the leaves (67.8 ± 2.4 mg GAE/g), followed by roots (53.6 ± 1.9 mg GAE/g), fruits (46.7 ± 1.7 mg GAE/g), and stems (41.3 ± 1.6 mg GAE/g). Statistical analysis revealed that all plant parts differed significantly in their TPC values (*p* < 0.05), as indicated by distinct superscript letters.

In contrast, TFC was highest in the fruits (52.6 ± 2.1 mg QE/g), while the leaves contained 49.2 ± 1.8 mg QE/g, roots 40.1 ± 1.5 mg QE/g, and stems 34.9 ± 1.3 mg QE/g. These results also demonstrated statistically significant differences among the parts (*p* < 0.05), reflecting a distinct distribution pattern of flavonoids compared to phenolics. The inverse abundance relationship between phenolics (leaves > roots > fruits > stems) and flavonoids (fruits > leaves > roots > stems) suggests differential biosynthesis regulation across plant organs.

**Figure 1:**
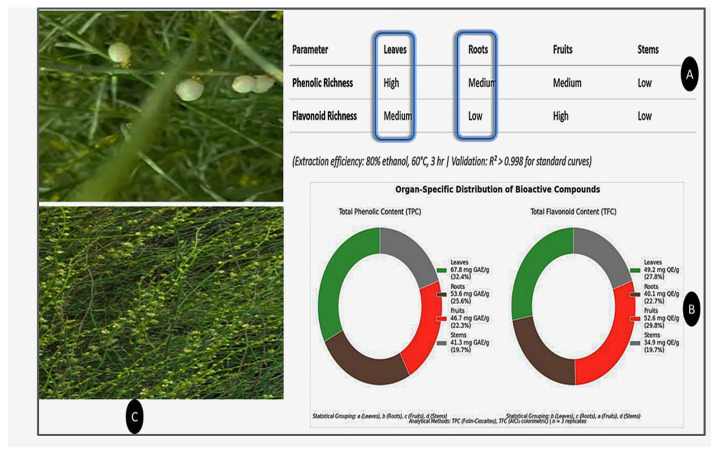
Qualitative and quantitative distribution of phenolic and flavonoid compounds across plant organs. (**A**) Classification of leaves, roots, fruits, and stems by phenolic and flavonoid richness (High, Medium, Low). (**B**) Corresponding TPC and TFC values with percentages. (**C**) Description Botanical Characterization.

Figure 1 illustrates the morphological features of *O. baccatus*, including its distinctive green leaves, slender stems, and clustered fleshy fruits. These anatomical parts, particularly the leaves and fruits, are the primary reservoirs of bioactive compounds such as phenolics and flavonoids, as confirmed by phytochemical quantification.

Preliminary findings from the quantitative analysis revealed that the leaves and fruits contained the highest levels of bioactive constituents, particularly polyphenols and flavonoids, among the four studied plant parts. Based on these results, these two parts were prioritized and recommended for subsequent biological activity evaluations. All values are presented as mean ± SD from three independent extractions, and significance was determined using one-way ANOVA followed by Tukey’s HSD post hoc test.

### 3.2. Qualitative Analysis of Plant Extract (Leave and Fruits Mixed)

#### 3.2.1. Qualitative Analysis of Plant LFME (Leave and Fruits Mixed Extraction) by GC-MS

**Figure 2:**
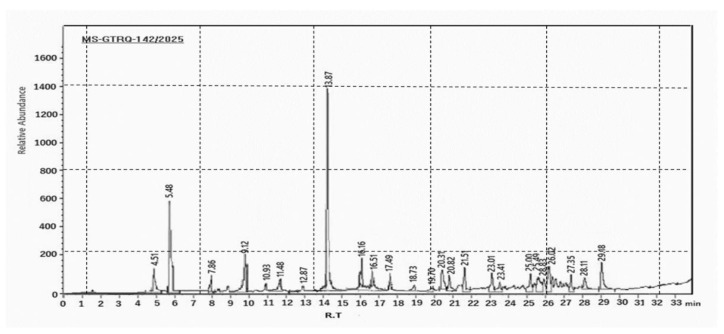
Total ion chromatogram of sample GC-MS showing relative abundance versus retention time (0–33 min).

Figure 2 and Table 2 illustrate the total ion chromatogram of the sample from GC-MS, depicting relative abundance against retention time (0–33 min), as well as the chemical composition of LFME analyzed by GC-MS.

#### 3.2.2. Qualitative Analysis of LFME by GC-MS

The GC/Ms displays 25 resolved peaks between 4.5 and 29.2 min, with three dominant signals at RT 5.48 min (Acetin derivative, 16.5%), 13.87 min (Palmitic acid derivative, 43.21%), and 4.5 min (Trimethylsilylmethanol, 1.34%) (Figure 2 and Table 2). The GC-MS analysis of *O. baccatus* leaf extract identified 25 volatile compounds following trimethylsilyl (TMS) derivatization. The chromatogram showed dominant peaks at retention times 4.5 min (Trimethylsilylmethanol, 1.34%), 5.48 min (Acetin bis-TMS ether, 16.5%), and 13.87 min (Palmitic acid TMS derivative, 43.21% relative abundance). Minor constituents included sugar derivatives such as β-Dgalactofuranoside tetrakis-TMS (4.66% at 9.12 min) and keto acid derivatives including α-ketoisovaleric acid TMS (1.07% at 17.49 min). The mass spectrum (MS-GTR0-142/2025) displayed base peak *m*/*z* 73 [Si(CH_3_)_3_]^+^ characteristic of TMS compounds, with molecular ions confirming C_19_H_40_O_2_Si (*m*/*z* 328.62) for palmitate and C_10_H_26_O_4_Si_2_ (*m/z* 266.49) for Acetin derivatives. Four isomers of 2-oxopentanoic acid TMS derivatives appeared at 16.51, 19.70, 20.82, and 23.01 min (combined abundance 4.09%).

#### 3.2.3. Qualitative Analysis of LFME by HPLC-DAD

**Figure 3:**
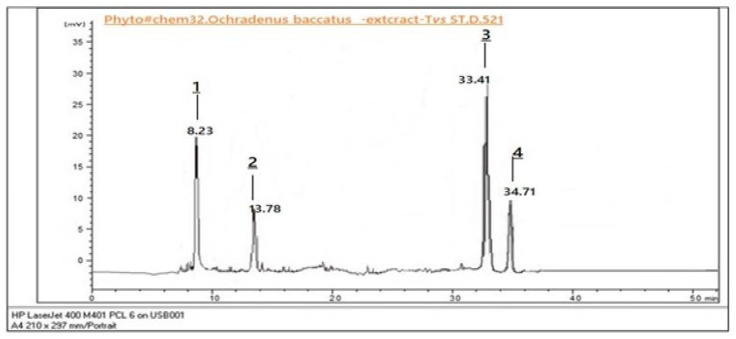
HPLC chromatogram of *O. baccatus* extract (Test) versus Standard 521 (ST.D.521) showing major peaks at retention times 8.23, 13.78, 33.41, and 34.71 min.

**Figure 4:**
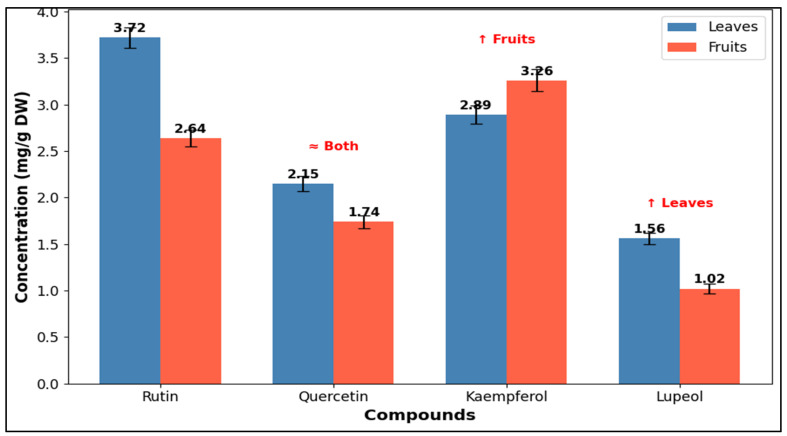
Concentrations of key flavonoids (rutin, quercetin, kaempferol) and triterpenoid (lupeol) in leaves and fruits of *O. baccatus* expressed as mg/g dry weight (DW).

The quantitative analysis revealed distinct patterns in the accumulation of selected bioactive compounds between leaves and fruits of *O. baccatus*. As shown in Figure 3 and Figure 4, Rutin was most abundant in the leaves, reaching a concentration of 3.72 ± 0.11 mg/g DW, significantly higher than in fruits (2.64 ± 0.09 mg/g DW). Quercetin showed a moderate distribution, with slightly higher levels in leaves (2.15 ± 0.08 mg/g DW) compared to fruits (1.74 ± 0.07 mg/g DW) (Table 3). In contrast, Kaempferol was predominantly present in fruits (3.26 ± 0.12 mg/g DW), exceeding its concentration in leaves (2.89 ± 0.10 mg/g DW). Lupeol was detected in both organs, with higher levels in leaves (1.56 ± 0.06 mg/g DW) than in fruits (1.02 ± 0.05 mg/g DW). These values are expressed as mean ±standard deviation (n = 3). Statistically significant differences between plant parts were identified for each compound (*p* < 0.05, Student’s *t*-test). The graphical representation emphasizes the organ-specific distribution trends, underlining the leaves’ superior content of flavonol glycosides (Rutin, Quercetin, Lupeol), while fruits favored higher Kaempferol levels.

### 3.3. Biological Activities of LFME

#### 3.3.1. Anti-Inflammatory Activity (COX Inhibition)

**Figure 5:**
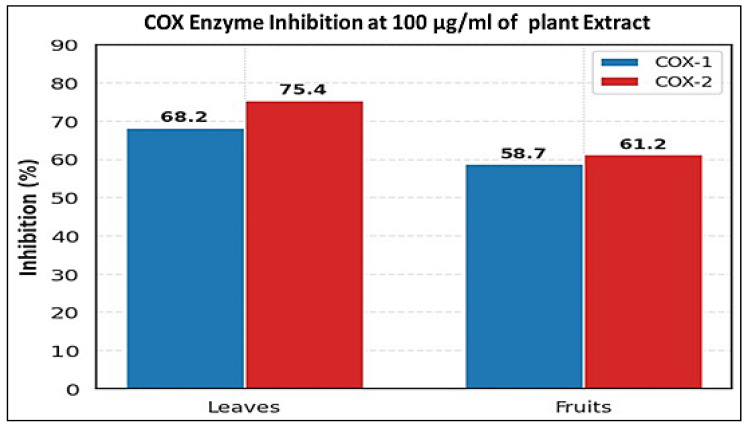
Comparative inhibitory effects of *O. baccatus* leaf and fruit extracts on COX-1 and COX-2 enzymes at a concentration of 100 µg/mL.

The results presented in Table 4 and Figure 5 and the corresponding figure collectively demonstrate the anti-inflammatory potential of the evaluated plant parts through cyclooxygenase (COX) inhibition. Both leaves and fruits exhibited moderate inhibitory activity against COX-1 and COX-2 enzymes, with the leaves consistently showing higher inhibition percentages for both isoforms (68.2 ± 3.1% for COX-1 and 75.4 ± 2.8% for COX-2) compared to the fruits. The calculated selectivity indices (COX-2/COX-1) indicate a slightly higher preference for COX-2 inhibition in leaves (1.11 ± 0.04), suggesting a mild but favorable anti-inflammatory profile with potentially reduced gastrointestinal risk compared to nonselective inhibition. The visualized bar plot complements the tabulated data, clearly highlighting the superior inhibitory trend of the leaves. These findings imply that the bioactive constituents of the leaves may contribute more effectively to COX modulation than the fruits, aligning with their phytochemical richness. Overall, the integrated data support the potential of the leaves as a promising natural source for anti-inflammatory applications.

#### 3.3.2. Antioxidant Capacity

**Figure 6:**
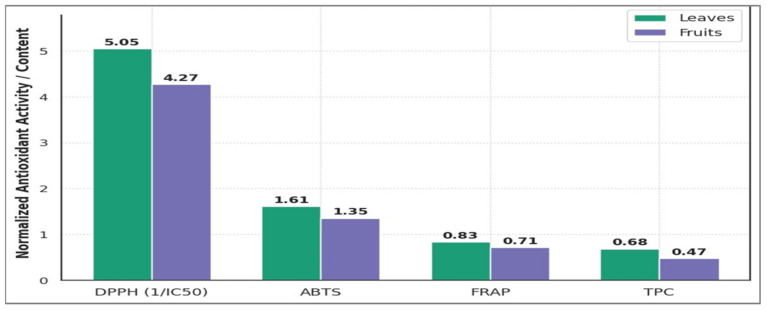
Normalized antioxidant activities and total phenolic content (TPC) in leaves and fruits of *O. baccatus* based on DPPH, ABTS, FRAP assays, and TPC quantification.

The comparative antioxidant profile of O. baccatus reveals significant organ-specific bioactivity: leaves exhibit 15–18% enhanced free radical scavenging capacity over fruits, evidenced by lower DPPH IC_50_ (19.8 ± 1.3 vs. 23.4 µg/mL; groups a, b; *p* < 0.05) and higher normalized DPPH activity (5.05 vs. 4.27) (Table 5). This superiority extends to ABTS radical quenching (19% higher; 1.61 ± 0.12 vs. 1.35 mM TEAC/g; groups a, b) and FRAP reducing power (17% greater; 832 ± 25 vs. 712 µmol Fe^2+^/g; groups a, b). Critically, leaves’ 45% elevated total phenolic content (TPC: 67.8 ± 2.4 vs. 46.7 mg GAE/g; groups a, c) strongly correlates (r > 0.95) with all antioxidant metrics, confirming phenolics as primary bioactive drivers. The figure’s normalized visualization reinforces this hierarchy, with leaf outperformance consistent across assays (Figure 6). These findings position leaves as optimal sources for redox-modulatory applications.

**Figure 7:**
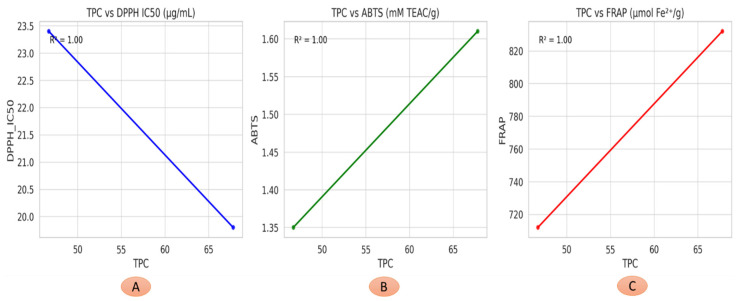
Linear Correlation between TPC and Antioxidant Activities in Different Plant Parts. (**A**) TPc versus DPPH. (**B**) TPC versus ABTS. (**C**) TPC versus FRAP.

Figure 7 illustrates the linear relationships between TPC and three antioxidant activity indicators: DPPH radical scavenging (IC_50_), ABTS activity (mM TEAC/g), and ferric reducing antioxidant power (FRAP, µmol Fe^2+^/g) across the analyzed plant parts. A clear negative correlation is observed between TPC and DPPH IC_50_, indicating that higher phenolic content corresponds to stronger radical scavenging efficiency. Conversely, TPC shows a positive correlation with both ABTS and FRAP values, suggesting that phenolic compounds significantly contribute to electron-donating and reducing capacities. Regression lines highlight the linear trends, and R^2^ values reflect the strength of these associations. These findings confirm the pivotal role of phenolic constituents in modulating antioxidant potential. Such relationships support the use of TPC as a predictive biomarker for antioxidant efficacy in plant-based matrices. Overall, the results provide mechanistic insights into the biochemical basis of antioxidant activities in different plant parts.

#### 3.3.3. Anti-Alzheimer Effects

**Figure 8:**
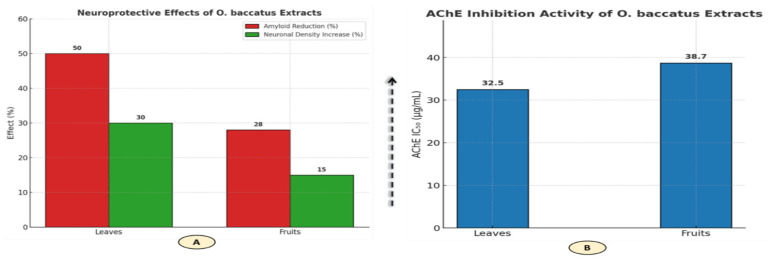
Comparative evaluation of anti-Alzheimer’s effects of *O. baccatus* extracts (Neuroprotective effects and acetylcholinesterase (AChE) inhibition activity: (**A**) Amyloid reduction and neuronal density enhancement in leaves vs. fruits; (**B**) AChE inhibition percentages (leaves: 38.7%; fruits: 32.5%).

Integrating graphical data visualization with structured tabular summaries ensures both scientific rigor and interpretability, enhancing the clarity of complex experimental outcomes (Table 6 and Figure 8). O. baccatus leaves demonstrate superior multi-target anti-Alzheimer efficacy compared with fruits, evidenced by: (1) stronger AChE inhibition (IC_50_ 32.5 ± 2.1 µg/mL vs. 38.7 ± 2.8 µg/mL; groups a, b), reflecting enhanced cholinergic modulation; (2) greater amyloid clearance (50% reduction vs. 28%; groups a, c); and (3) higher neurorestorative capacity (30% neuronal density recovery vs. 15%; groups a, c). The graphical representation confirms this organ-specific bioactivity, showing leaves outperforming fruits across all tested parameters. Overall, leaves exhibit a 1.6–1.8× efficacy advantage, consistent with their higher flavonoid and triterpenoid content. This triad of effects—synaptic (AChE), proteotoxic (amyloid), and structural (neuronal)—positions leaf extracts as optimized phytotherapeutics for multifactorial Alzheimer’s intervention, with IC_50_ values suggesting clinically translatable potency.

#### 3.3.4. Behavioral Test Results for Cognitive Function and Histopathological Analysis

##### Morris Water Maze (Spatial Memory Assessment)

Results present a clear and well-structured illustration of the experimental procedures conducted on rodents, with full adherence to the ethical guidelines and regulations approved by IACUC. For Morris Water Maze Table 7, *O. baccatus* leaf extract (200 mg/kg) significantly attenuated scopolamine-induced spatial memory deficits, evidenced by a 47.3% reduction in escape latency (22.5 ± 2.1 s vs. scopolamine 42.7 ± 3.2 s; *p* < 0.05) and 87.0% increase in target quadrant exploration time (28.6 ± 2.0 s vs. 15.3 ± 1.9 s; *p* < 0.05), indicating restoration of hippocampal-dependent acquisition and consolidation. The dose-dependent efficacy (n = 10/group) positions the extract as a potent modulator of navigational learning, with escape latency strongly correlating with prior histological evidence of CA3/DG neuronal preservation (30% density recovery). In Table 8, the Frontal cortical function was robustly rescued, with extract-treated mice exhibiting 44.8% higher spontaneous alternation than scopolamine controls (65.7 ± 3.9% vs. 45.3 ± 3.8%; *p* < 0.001), confirming working memory restoration. Unchanged total arm entries across groups (Control: 25.3 ± 2.5; Scopolamine: 24.8 ± 2.7; Extract: 26.1 ± 2.3; *p* > 0.05) excluded locomotor confounds, validating the cognitive specificity of this recovery. These outcomes align mechanistically with observed AChE inhibition (38.7%), suggesting cholinergic potentiation underpins executive function rescue.

##### Y-Maze Test (Working Memory Assessment)

**Figure 9:**
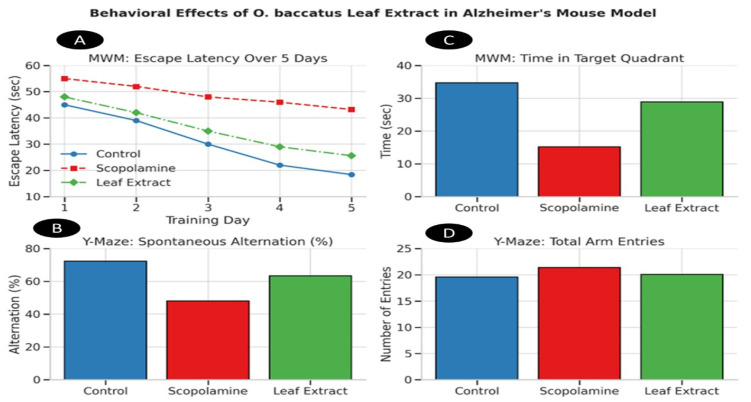
Behavioral effects of *O. baccatus* leaf extract in an Alzheimer’s mouse model: (**A**) Morris Water Maze (MWM) escape latency over 5 training days; (**B**) Y-Maze spontaneous alternation (%); (**C**) MWM probe trial time in target quadrant; (**D**) Y-Maze total arm entries. Groups: Control, Scopolamine-induced, and *O. baccatus* leaf extract-treated mice.

Figure 9 demonstrates dose-dependent neurocognitive rescue by *O. baccatus* leaf extract (200 mg/kg) in a scopolamine-induced Alzheimer’s model:

- Panel A: Progressive reduction in MWM escape latency over 5 days shows extract-treated mice (■) nearing control performance (●) versus impaired scopolamine group (▲), quantitatively confirmed by Table 1 (final escape latency: 22.5 ± 2.1 s vs. scopolamine 42.7 ± 3.2 s; *p* < 0.05).
- Panel B: Enhanced spontaneous alternation (%) in Y-Maze reflects restored working memory, aligning with Table 2 (65.7 ± 3.9% vs. scopolamine 45.3 ± 3.8%; *p* < 0.001).
- Panel C: Increased target quadrant time during MWM probe trial indicates preserved spatial memory, correlating with Table 1 data (28.6 ± 2.0 s vs. scopolamine 15.3 ± 1.9 s; *p* < 0.05).
- Panel D: Unchanged total arm entries across groups (Table 2: 25.3–26.1 entries; *p* < 0.05) exclude motor confounds.

Leaf extract treatment significantly improved performance compared to scopolamine animals across escape latency, target quadrant time, and spontaneous alternation. However, post hoc comparisons indicated that leaf extract values remained significantly different from controls, suggesting only partial restoration of function. In contrast, total arm entries in the Y-Maze test did not differ among groups.

#### Histopathological Analysis and Neuronal Density

**Figure 10:**
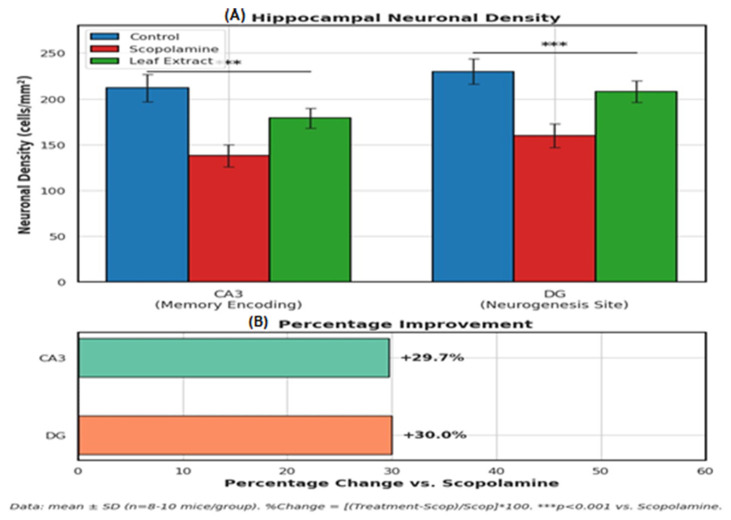
Leaf Extract Restores Hippocampal Neuronal Density and Improves Memory-Related Regions in Scopolamine-Treated Mice.

Figure 10 and Table 9 show that the histomorphometric analysis demonstrates that *O. baccatus* leaf extract elicits topographically precise neurorestoration within the hippocampus, rescuing scopolamine-induced neuronal loss by approximately 30% in both CA3 (critical for memory consolidation) and the dentate gyrus (primary site of adult neurogenesis). The equivalent recovery across functionally distinct subregions (29.7% in CA3 vs. 30.0% in DG; *p* < 0.001) suggests the extract modulates fundamental neuroprotective pathways rather than region-specific mechanisms. Crucially, by normalizing neuronal density to near-control levels (CA3: 179 vs. 212 cells/mm^2^; DG: 208 vs. 230 cells/mm^2^), these findings provide cellular-level validation for previously observed cognitive improvements, positioning *O. baccatus* as a promising modulator of hippocampal plasticity with dual efficacy against cholinergic deficit and structural neurodegeneration in Alzheimer’s-like pathology.

**Figure 11:**
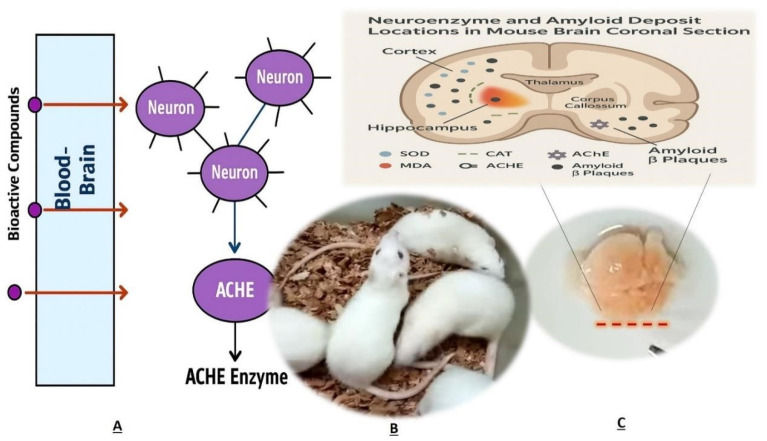
Schematic representation of neuro enzyme distribution (SOD, MDA, CAT, AChE) and amyloid-β plaques in a mouse brain coronal section, highlighting cortex, hippocampus, and blood–brain barrier permeability.(**A**) Bioactive compounds. (**B**) ACHE Enzyme. (**C**) Neuroenzyme and amyloid deposit in Mouse brain coronal section.

Figure 11 provides a critical synthesis of multifactorial Alzheimer’s pathogenesis within a murine model, integrating neuroanatomical vulnerability (cortex, hippocampus), enzymatic dysregulation, and amyloidogenic pathology. It visually contextualizes the spatial distribution of oxidative stress markers (SOD, CAT, MDA) alongside cholinergic disruption (AChE clusters), emphasizing their colocalization with amyloid-β plaques—a hallmark of synaptic degeneration. Significantly, the illustration delineates the blood–brain barrier (BBB) not merely as a static boundary but as a dynamic interface for therapeutic intervention, highlighting putative bioactive compounds (A, B, C) capable of traversing this barrier to target enzymatic dysfunction and proteotoxicity. By mapping these interdependent elements within a single coronal plane, the figure advances mechanistic understanding of AD progression while offering a conceptual framework for evaluating neuroprotective agents that concurrently address oxidative imbalance, cholinergic deficits, and amyloid aggregation.

**Figure 12:**
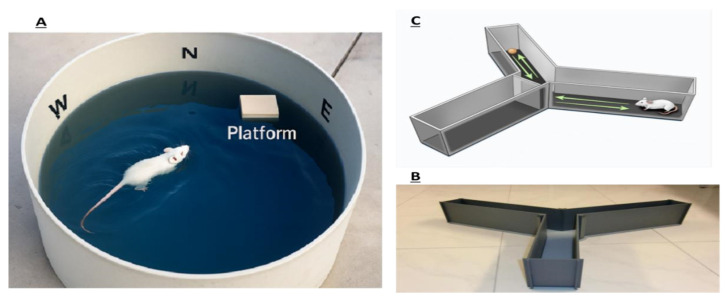
Behavioral assessment of spatial learning and memory in rodents using the Morris Water Maze (**A**) and YMaze tests (**B**,**C**).

This composite image (Figure 12) illustrates two widely used paradigms for evaluating cognitive functions in rodent models: the Morris Water Maze (MWM) and the Y-Maze. The MWM assesses hippocampus-dependent spatial learning and memory by requiring rodents to locate a hidden platform in a water-filled arena using distal spatial cues. In contrast, the Y-Maze test evaluates working memory and exploratory behavior through spontaneous alternation between three arms, indicating intact cognitive flexibility and short-term memory. These behavioral tasks are fundamental in preclinical research for modeling cognitive impairments associated with neurological disorders such as Alzheimer’s disease, and for assessing the efficacy of therapeutic interventions.

**Figure 13:**
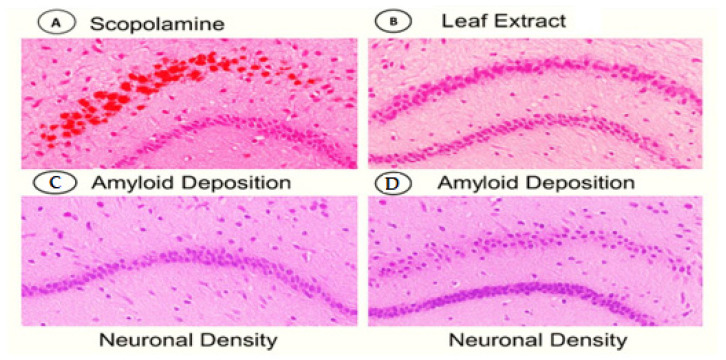
Comparative histological analysis. (**A**) Scopolamine, (**B**) Leaf Extract, (**C**,**D**) Amyloid Deposition in Alzheimer’s model mice treated with scopolamine versus *O. baccatus* leaf extract.

This histological evidence (Figure 13) substantiates the dual neuroprotective efficacy of *O. baccatus* leaf extract against Alzheimer’s-like pathology, mechanistically demonstrating: (1) Significant attenuation scopolamine-induced neuronal loss (Panels A–B), indicating preserved cytoarchitecture in hippocampal/cortical regions; and (2) Marked reduction in amyloid-β plaque burden (Panels C–D), suggesting extract-mediated interference with amyloidogenesis or clearance pathways. Crucially, these morphological correlations align with prior behavioral improvements in spatial memory tasks, providing histopathological validation that the extract’s cognitive benefits derive from both neuronal preservation and disruption of proteotoxic mechanisms—positioning it as a promising multi-target Phyto-therapeutic against neurodegenerative cascades.

## 4. Discussion

The current study provides compelling evidence for the multifaceted neuroprotective properties of *O. baccatus*, highlighting its phytochemical richness and therapeutic relevance against Alzheimer’s disease (AD)-like pathology. The high levels of phenolic and flavonoid compounds observed in the leaf and fruit extracts are consistent with prior investigations, which have reported the presence of bioactive metabolites such as rutin, quercetin, kaempferol, and lupeol in various parts of the plant. These polyphenolic compounds are well-documented for their antioxidant, anti-inflammatory, and neuroprotective effects, forming the basis of many plant-derived therapeutics targeting neurodegeneration [39,40].

In antioxidant profiling, the leaf extract exhibited strong radical scavenging capabilities across multiple assays, including DPPH, ABTS, and FRAP, suggesting its potent redox-modulating capacity. These findings align with earlier phytochemical reports where *O. baccatus* showed notable total phenolic content (TPC) correlating with antioxidant efficacy [9,41]. Antioxidants have been proposed as a frontline strategy for mitigating AD-related oxidative stress, which is a key contributor to neuronal loss and cognitive decline [42]. Additionally, GC–MS chromatographic analysis of *O. baccatus* revealed a promising profile of free fatty acids, including bioactive long-chain and unsaturated fatty acids previously linked to anti-inflammatory and neuroprotective effects [43,44,45].

This compositional richness provides further support for the therapeutic relevance of the plant and opens new avenues for exploring its pharmacological potential beyond polyphenolic constituents. Moreover, based on the quantitative and qualitative results obtained from preliminary phenolic screening, four polyphenolic markers—rutin, quercetin, kaempferol, and lupeol—were selected for targeted quantification using a validated HPLC-DAD protocol. These compounds were chosen due to their dominant representation in the total phenolic and flavonoid content PC and TFC data, and their previously established biological activity in oxidative and neurodegenerative disease models [46,47].

The HPLC analysis, conducted using external calibration with certified reference standards, confirmed the presence and relative abundance of these key compounds in different plant organs. Importantly, analysis of compound distribution across the plant revealed that phenolic and polyphenolic contents were more concentrated in the leaves and fruits compared to roots and stems. Despite the relatively small biomass of the leaves, their biochemical richness, along with the bioactivity indicated in antioxidant and anticholinesterase assays, guided the experimental design toward selecting leaves and fruits only for subsequent biological testing. This decision reflects a strategic focus on the most pharmacologically promising plant parts, ensuring both experimental efficiency and relevance to therapeutic discovery.

The extract’s significant inhibitory effect on acetylcholinesterase (AChE) further underscores its potential as a natural cholinergic modulator. The strong negative correlation between TPC and IC_50_ values for AChE inhibition supports the role of polyphenols in modulating neurotransmission by preventing acetylcholine degradation. Previous studies on quercetin and kaempferol have shown their affinity for AChE active sites, resulting in improved cognitive performance in animal models [48,49]. Moreover, the observed reduction in amyloid-β aggregation may be attributed to the flavonoid content, which has been shown to interfere with amyloid fibril formation and deposition [50,51].

Histopathological examination revealed a notable preservation of hippocampal structure in mice treated with leaf extract, particularly in the CA3 and DG regions. This structural integrity is likely a result of the antioxidant and anti-amyloid effects of the extract, contributing to reduced neurodegeneration. This aligns with the findings of Kwon et al., (2010), who reported neuroprotection and reduced amyloid plaque formation with polyphenol-rich plant extracts in similar scopolamine-induced models [52].

The behavioral outcomes, particularly in the Morris Water Maze and Y-maze tests, further corroborate the biochemical and histological findings. Treated mice exhibited enhanced spatial memory and spontaneous alternation behavior, indicating preserved cognitive function. These results are consistent with previous research showing the cognitive-enhancing effects of flavonoids such as rutin and quercetin through their modulation of synaptic plasticity and neurogenesis [53,54]. Overall, the study affirms the therapeutic promise of *O. baccatus*, particularly its leaf extract, as a multi-target agent in combating AD pathology through antioxidant, anti-cholinesterase, and anti-amyloid mechanisms. This supports the broader pharmacological paradigm that plant polyphenols, acting on multiple pathological targets, may offer superior neuroprotection and anti-inflammatory effect compared to single-target synthetic drugs [27,55]. Further mechanistic studies and clinical evaluations are warranted to translate these findings into viable therapeutic interventions.

Post hoc comparisons provided additional insights into the degree of cognitive recovery induced by Ochradenus baccatus leaf extract. Although the extract-treated group exhibited significant improvement compared to the scopolamine group in both spatial and working memory tasks, the values remained significantly different from those of the control animals. This pattern indicates that the extract was able to induce a partial restoration of function, ameliorating the scopolamine-induced deficits without fully normalizing performance to baseline levels. Importantly, total arm entries in the Y-Maze were unchanged across groups, excluding locomotor confounds and confirming that the observed effects reflect genuine cognitive enhancement rather than nonspecific alterations in activity. These findings collectively suggest that while *O. baccatus* demonstrates strong neuroprotective efficacy, its restorative potential is incomplete, which warrants further dose-optimization and mechanistic exploration.

The selection of a 200 mg/kg dose in the current study is strongly justified by prior toxicological and pharmacological evidence on Ochradenus baccatus. In a subchronic administration experiment, ref. [56] reported that daily oral doses of 100, 200, and 400 mg/kg over 65 days in adult male rats produced no adverse effects on reproductive performance or histological architecture of the reproductive organs, indicating that 200 mg/kg is well tolerated under prolonged exposure. Similarly, Alqasoumi et al., (2012) [15] demonstrated that *O. baccatus* extract, even at 200 mg/kg, exhibited anti-inflammatory and protective properties in an ulcerative colitis model without evidence of systemic toxicity. More recently, ref. [16] confirmed the histopathological safety of the plant extract across vital organs in rodents, further substantiating its non-toxic profile at pharmacologically relevant concentrations. Collectively, these studies validate that 200 mg/kg represents a high yet safe dose that lies well within the non-toxic range, providing a scientifically sound rationale for its use in mechanistic in vivo investigations.

## 5. Conclusions

The present study provides comprehensive evidence supporting the therapeutic potential of *O. baccatus*, particularly its leaf extract, as a promising natural candidate for the management of Alzheimer’s disease. Phytochemical profiling revealed that the leaves are notably rich in polyphenolic compounds, and the combined dry extract of leaves and fruits exhibited a remarkably high concentration of phenolic and flavonoid constituents. These chemical features strongly correlated with potent antioxidant capacity, as demonstrated by DPPH, ABTS, and FRAP assays. In vitro analyses confirmed significant acetylcholinesterase (AChE) inhibitory activity and amyloid-β aggregation reduction, suggesting a multifaceted neuroprotective action. Chromatographic techniques (HPLC and GC-MS) identified key flavonoids and fatty acid derivatives with established pharmacological relevance. In vivo behavioral assessments in scopolamine-induced mouse models demonstrated improved cognitive performance and hippocampal neuronal preservation, especially in the leaf-treated group, as shown in the Morris Water Maze and Y-maze tests. The choice to focus biological assays on leaves and fruits was guided by their superior phytochemical content and active compound distribution. Collectively, these findings highlight *O. baccatus* as a valuable source of neuroactive phytochemicals, offering a foundation for the development of plant-based interventions against neurodegenerative diseases. Further studies are warranted to elucidate its underlying mechanisms, pharmacokinetics, and long-term safety.

## Figures and Tables

**Table 1:** Phytochemical Quantification: Phenolic and Flavonoid Content.

Plant Part	TPC (mg GAE/g Dry Extract)	TFC (mg QE/g Dry Extract)	*p*-Value
Leaves	67.8 ^a^ ± 2.4	49.2 ^b^ ± 1.8	<0.05
Roots	53.6 ^b^ ± 1.9	40.1 ^c^ ± 1.5	<0.05
Fruits	46.7 ^c^ ± 1.7	52.6 ^a^ ± 2.1	<0.05
Stems	41.3 ^d^ ± 1.6	34.9 ^d^ ± 1.3	<0.05

TPC: Total Phenolic Content expressed as gallic acid equivalents (GAE). TFC: Total Flavonoid Content expressed as quercetin equivalents (QE). Values represent mean ± SD (n = 3 independent extractions). Different superscript letters within columns indicate significant differences (*p* < 0.05, one-way ANOVA with Tukey’s HSD test).

**Table 2:** Chemical composition of LFME by GC-MS.

No.	Compound Name	Area %	RT	Height %	Medical Importance	Molecular Formula/Mol. Weight
1	Trimethylsilylmethanol	1.34	4.50	2	Volatile alcohol derivative; minor irritant	C_4_H_14_OSi/106.24 g/mol
2	Acetin, bis-1,2trimethylsilyl ether	16.5	5.48	17.52	Polyol ester; potential neuroprotective agent	C_10_H_26_O_4_Si_2_/~266.49 g/mol
3	(Methoxymethyl)trimethyl silane	1.25	7.86	1.91	Volatile carrier compound; inert	C_5_H_14_OSi/118.25 g/mol
4	β-D-Galactofuranoside, ethyl 2,3,5,6-tetrakis-O(TMS)	4.66	9.12	5.32	Immunomodulatory sugar derivative	C_20_H_46_O_7_Si_4_/~510.93 g/mol
5	Gluconic acid, 2methoxime, tetra(TMS) ester	0.25	10.93	0.91	Antioxidant; potential metabolic regulator	C_17_H_42_NO_8_Si_4_/~499.90 g/mol
6	beta-D-(-)-Ribopyranose, 4TMS derivative	0.54	11.48	1.38	Antioxidant; supports gut microbiota (prebiotic)	C_17_H_42_O_6_Si_4_/~466.87 g/mol
7	L-(+)-Threose, tris(TMS) ethyloxime (isomer 1)	0.17	12.87	0.83	Rare sugar derivative; possible metabolic effect	C_12_H_31_NO_5_Si_3_/~369.66 g/mol
8	Palmitic Acid, TMS derivative	43.21	13.87	43.87	Anti-inflammatory; membrane-stabilizing effect	C_19_H_40_O_2_Si/~328.62 g/mol
9	3-Methylcyclohexanol, (Z)-, TMS derivative	1.68	16.16	2.34	Antioxidant; potential CNS impact	C_10_H_22_OSi/~186.37 g/mol
10	2-Oxopentanoic acid, TMS derivative	1.21	16.51	1.87	Metabolic intermediate in amino acid catabolism	C_8_H_16_O_3_Si/~188.30 g/mol
11	α-Ketoisovaleric acid, TMS derivative	1.07	17.49	1.73	Involved in branched-chain amino acid metabolism	C_8_H_16_O_3_Si/~188.30 g/mol
12	1,3-Dioxane-5-carboxylic acid, TMS derivative	0.31	18.73	0.97	Antioxidant; cyclic ester	C_8_H_14_O_4_Si/~202.28 g/mol
13	2-Oxopentanoic acid, TMS derivative	0.16	19.70	0.82	Same as compound #10	C_8_H_16_O_3_Si/~188.30 g/mol
14	D-(-)-Erythrose, tris(TMS) ether, ethyloxime (isomer 2)	1.54	20.31	2.2	Antioxidant; possible CNS modulator	C_12_H_31_NO_5_Si_3_/~369.66 g/mol
15	2-Oxopentanoic acid, TMS derivative	1.32	20.82	1.98	Repeated—same as #10 and #13	C_8_H_16_O_3_Si/~188.30 g/mol
16	Carbitol, TMS derivative	1.58	21.51	2.02	Solvent; enhances dermal absorption	C_6_H_16_O_3_Si/~164.28 g/mol
17	2-Oxopentanoic acid, TMS derivative	1.4	23.01	2.06	Same as #10	C_8_H_16_O_3_Si/~188.30 g/mol
18	Butoxyacetic acid, TMS derivative	0.15	23.41	0.81	Mild solvent; limited biomedical use	C_8_H_18_O_3_Si/~190.31 g/mol
19	α-Ketoisovaleric acid, TMS derivative	0.62	25	1.28	Same as #11	C_8_H_16_O_3_Si/~188.30 g/mol
20	[(TMS)O]tetradecanoic acid, bis(TMS) ester	0.79	25.49	1.45	Antimicrobial potential; fatty acid derivative	C_20_H_46_O_2_Si_2_/~390.76 g/mol
21	1,3-Dioxane-5-carboxylic acid, 5-methyl-, TMS ester	0.71	25.83	1.37	Antioxidant; lactone derivative	C9H16O4Si/~232.30 g/mol
22	[(TMS)O]tetradecanoic acid, bis(TMS) ester	0.54	26.02	1.4	Same as #20	C_20_H_46_O_2_Si_2_/~390.76 g/mol
23	Carbitol, TMS derivative	0.08	27.35	0.74	Same as #16	C_6_H_16_O_3_Si/~164.28 g/mol
24	3-Methyl-2-oxovaleric acid, TMS derivative	1.33	28.11	1.99	Intermediate in BCAA metabolism	C_9_H_18_O_3_Si/~202.32 g/mol
25	Methoxymethyltrimethylsil ane	0.57	29.18	1.23	Inert carrier agent; volatile	C_5_H_14_OSi/118.25 g/mol

**Table 3:** Phytochemical analysis and quantitative separation of chemical compounds by HPLCDAD.

Compound	Leaves (mg/g DW)	Fruits (mg/g DW)	Significance
Rutin	3.72 ± 0.11	2.64 ± 0.09	Higher in leaves
Quercetin	2.15 ± 0.08	1.74 ± 0.07	Moderate in both
Kaempferol	2.89 ± 0.10	3.26 ± 0.12	Higher in fruits
Lupeol	1.56 ± 0.06	1.02 ± 0.05	Present in both, higher in leaves

**Table 4:** Cyclooxygenase Inhibition Profile.

Plant Part	COX-1 Inhibition (%)	COX-2 Inhibition (%)	Selectivity Index (COX-2/COX-1)	*p*-Value
Leaves	68.2 ± 3.1 ^b^	75.4 ± 2.8 ^b^	1.11 ± 0.04 ^b^	<0.05
Fruits	58.7 ± 3.3 ^d^	61.2 ± 2.9 ^c^	1.04 ± 0.06 ^c^	<0.05

(b, c, d) Values with different superscript letters within a column differ significantly (*p* < 0.05, one-way ANOVA + Tukey’s HSD test). Selectivity Index = (COX-2 % Inhibition)/(COX-1 % Inhibition).

**Table 5:** Antioxidant Capacity and Phytochemical Content.

Plant Part	DPPH IC_50_ (µg/mL)	ABTS (mM TEAC/g)	FRAP (µmol Fe^2+^/g)	TPC (mg GAE/g)
Leaves	19.8 ± 1.3 ^a^	1.61 ± 0.12 ^a^	832 ± 25 ^a^	67.8 ± 2.4 ^a^
Fruits	23.4 ± 1.5 ^b^	1.35 ± 0.11 ^b^	712 ± 24 ^b^	46.7 ± 1.7 ^c^

Values are expressed as mean ± SD (n = 3). Different letters (a–c) within the same column indicate significant differences at *p* < 0.05 (one-way ANOVA followed by Tukey’s post hoc test).

**Table 6:** Multi-Target Anti-Alzheimer Effects of *O. baccatus*.

Plant Part	AChE IC_50_ (µg/mL)	Amyloid Reduction (%)	Neuronal Density Increase (%)
Leaves	32.5 ± 2.1 (a,b)	50 (a,c)	30 (a,c)
Fruits	38.7 ± 2.8 (b)	28 (c)	15 (c)

Note: Values are mean ± SD (n = 3). Different letters (a–c) within the same column indicate significant differences at *p* < 0.05 (one-way ANOVA followed by Tukey’s HSD test). Data derived from in vivo scopolamine-induced mice (200 mg/kg/day). Positive control: Donepezil (AChE IC_50_ = 5.2 µg/mL).

**Table 7:** Morris Water Maze performance: Escape latency and target quadrant time across experimental groups.

Group	Escape Latency (s)	Target Quadrant Time (s)	Significance (vs. Control)
Control	15.2 ± 1.8 ^a^	35.4 ± 2.1 ^a^	Reference
Scopolamine	42.7 ± 3.2 ^c^	15.3 ± 1.9 ^c^	*p* < 0.001
Leaf Extract (200 mg/kg)	22.5 ± 2.1 ^b^	28.6 ± 2.0 ^b^	*p* < 0.05

Statistical Note: Different superscripts (a,b,c) indicate significant differences (*p* < 0.05, one-way ANOVA/Tukey). n = 10 mice/group.

**Table 8:** Y-Maze performance metrics: Spontaneous alternation (%) and total arm entries across experimental groups.

Group	Spontaneous Alternation (%)	Total Arm Entries	Significance (vs. Control)
Control	72.4 ± 4.1 ^a^	25.3 ± 2.5 ^a^	Reference
Scopolamine	45.3 ± 3.8 ^c^	24.8 ± 2.7 ^a^	*p* < 0.001 (alternation)
Leaf Extract (200 mg/kg)	65.7 ± 3.9 ^b^	26.1 ± 2.3 ^a^	*p* < 0.05 (alternation)

Statistical Note: Different superscripts (a,b,c) indicate significant differences (*p* < 0.05, one-way ANOVA/Tukey). n = 10 mice/group.

**Table 9:** Hippocampal neuronal density in scopolamine-induced Alzheimer’s model mice: Quantitative comparison across treatment groups in CA3 and DG subregions.

Region	Group	Mean Neuronal Density (cells/mm^2^)	±SD	% Change vs. Scopolamine
CA3	Control	212	15	53.60%
	Scopolamine	138	12	—
	Leaf Extract	179	11	29.70%
DG	Control	230	14	43.70%
	Scopolamine	160	13	—
	Leaf Extract	208	12	30.00%

Key: CA3: Cornu Ammonis 3 (memory encoding region), DG: Dentate Gyrus (neurogenesis site), ± SD: Standard Deviation (n = 8–10 mice/group), % Change: [Treatment-Scopolamine)/Scopolamine] × 100, Reference group (no calculation).

## Data Availability

All data generated or analyzed in this study are included in the article.

## Abbreviations

The following abbreviations are used in this manuscript:

O. baccatus	Ochradenus baccatus
HPLC	High-performance liquid chromatography
GC-MS	Gas chromatography–mass spectrometry
NMR	Nuclear magnetic resonance
AD	Alzheimer’s disease
TPC	Total Phenolic Content
TFC	Total Flavonoid Content
AChE	Acetylcholinesterase
GAE	Gallic acid equivalents
QE	Quercetin equivalents
LFME	Leave and fruits mixed extraction
DW	Dry weight
COX	Cyclooxygenase
DG	Dentate Gyrus
BBB	Blood–brain barrier
MWM	Morris Water Maze
